# Effect of Cr on the Microstructure and Mechanical Properties of Cu-Ni-Si Alloys

**DOI:** 10.3390/ma19071353

**Published:** 2026-03-29

**Authors:** Hu Wang, Wanyu Wang, Zhongping Chen

**Affiliations:** Chinalco Science and Technology Research Institute Co., Ltd., Beijing 102209, China; hu_wang625@chinalco.com.cn (H.W.); zhongping_chen@chinalco.com.cn (Z.C.)

**Keywords:** Cu-Ni-Si alloy, Cr addition, soft and hard micro-zones, strength

## Abstract

A systematic study was conducted on the influence of Cr on the property evolution and precipitation behavior of Cu-Ni-Si alloys. Results indicate that Cu-Ni-Si alloys containing 0.33 at% Cr exhibit superior mechanical properties after three-stage cryogenic rolling and aging, achieving a tensile strength of up to 862 MPa. The addition of Cr induces competitive precipitation behavior between Cr and Ni for Si. It promotes the precipitation of Cr_3_Si phases at various scales while suppressing the formation of Ni_3_Si phases. Concurrently, it enhances the precipitation of fine nanoscale precipitation-hardening phases Ni_2_Si, optimizing the alloy’s precipitation hardening effect. Furthermore, the addition of Cr suppresses dislocation annihilation. The formation of finer precipitates pins the dislocations introduced during cryogenic rolling and impedes their motion, thereby enhancing the alloy’s strength and hardness. The alternating and staggered distribution of soft and hard microzones in the Cr-containing alloy results in more uniform overall properties of the sample. However, the reduced proportion of soft microzones slightly decreases the alloy’s electrical conductivity.

## 1. Introduction

Cu-Ni-Si alloys are indispensable in electronics, information technology, new energy vehicles, and aerospace owing to their outstanding electrical conductivity, high strength, and superior stress relaxation resistance. They are extensively used in precision components such as connectors, integrated circuit lead frames, and relay reed switches [1,2,3]. As the representative high-strength, medium-conductivity copper alloy, it typically exhibits a tensile strength of 600–860 MPa and an electrical conductivity of 30–60% IACS [4,5]. This precipitation-hardening alloy faces the core challenge of simultaneously enhancing strength and conductivity. Microalloying combined with thermomechanical processing offers an effective means to mitigate conductivity loss while improving strength, thereby achieving an optimized strength–conductivity balance. This strategy provides a critical pathway for the development of high-strength, high-conductivity copper alloys.

In the study of microalloying in the Cu-Ni-Si system, multiple elements have been employed to promote precipitation and purify the matrix. Al suppresses the coarsening of nanoscale precipitation phases, forms precipitation phases containing Ni and Al, enhances alloy strength and electrical conductivity, and provides stress relaxation resistance [6,7]. Zr addition forms coarse Ni_2_SiZr phases, accelerating the precipitation rate of the alloy. During aging, Zr accelerates the diffusion of solute atoms, promoting the coarsening of precipitates, thereby reducing alloy strength but improving electrical conductivity [8,9]. Co substitution for Ni forms δ-(Ni, Co)_2_Si phases, significantly enhancing alloy strength and electrical conductivity [10,11]. Ti refines grain size and improves ductility but promotes Ni_2_Si phase growth and coarsening, facilitating lamellar structure formation and reducing alloy strength [12,13]. Mg simultaneously enhances strength and stress relaxation resistance by dragging dislocations and reducing the spacing between δ-Ni_2_Si phases [14]. In contrast, Cr is considered one of the most promising microalloying elements due to its proximity to Cu in the periodic table, resulting in similar atomic radii and shear moduli [15]. Appropriate Cr addition not only promotes the precipitation of the Ni_2_Si phase but also forms fine, thermally stable Cr_3_Si phases. These phases effectively inhibit dislocation motion and grain growth while improving electrical conductivity by reducing the number of solute atoms [16,17,18]. Wang et al. [19] reported that trace amounts of Cr exert a dual influence on alloy aging, facilitating the attainment of high strength while causing only a negligible decrease in electrical conductivity. Wang et al. [20] found that Cr and Cu have similar atomic radii and shear moduli, readily forming supersaturated solid solutions. This promotes the precipitation of the Ni_2_Si second phase and increases the number of precipitated phases, leading to improvements in both strength and electrical conductivity. Rdazwski Z [21] found that the content of Cr_3_Si and Ni_2_Si phases is optimized at a Cr content of 0.8%. Cr_3_Si exhibits a small particle size, high dissolution temperature, and strong high-temperature stability, effectively inhibiting dislocation motion and grain growth. Furthermore, the Si content in the matrix increases after Cr_3_Si precipitation, enhancing electrical conductivity. Despite these benefits, the mechanism of Cr’s role in Cu-Ni-Si alloys, particularly its synergistic regulation of microstructural evolution and properties, remains understudied.

Beyond composition control, deformation heat treatment processes also exert a critical influence on the microstructure and properties of alloys. Traditional strip preparation methods often enhance strength at the expense of electrical conductivity. How to maximize electrical conductivity while maintaining high strength remains a key research focus in this field [22]. Theoretically, promoting the complete precipitation of nano-phases from the matrix while ensuring nanoscale precipitation and high dislocation density is an effective approach to achieve synergistic improvements in strength and electrical conductivity [23]. Cryogenic rolling suppresses dislocation slip and restricts dislocation movement. It enhances dislocation storage capacity by introducing shear bands while simultaneously fracturing grains at the nanoscale under shear stress, thereby promoting precipitation-phase nanostructuring [24,25]. Liao et al. [26] compared the effects of room-temperature rolling and cryogenic rolling on the properties of Cu-2.8Ni-0.6Si-0.1Mg alloy. They found that the C70250 copper alloy subjected to large-deformation cryogenic rolling exhibited higher dislocation density. This occurs because cryogenic rolling freezes dislocation motion, hindering dislocation slip. Shear bands formed during cold rolling increase dislocation storage capacity, shear stress breaks grains down to nanoscale dimensions, and high lattice distortion transforms the matrix toward an amorphous structure, promoting solute atom diffusion and recrystallization.

This study investigates the effects of Cr on the microstructure and properties of Cu-Ni-Si alloys. Multi-pass cryogenic rolling combined with heat treatment is employed to develop a multiscale precipitate-dense, fine-grained microstructure with high dislocation density. The role of Cr in governing structural evolution and property enhancement is elucidated, aiming to produce high-strength, high-conductivity Cu-Ni-Si-Cr alloys. The findings provide theoretical and experimental foundations for the development of next-generation high-strength, medium-conductivity copper alloys.

## 2. Materials and Methods

### 2.1. Material Preparation

Cu-Ni-Si and Cu-Ni-Si-Cr alloys were prepared using a vacuum medium-frequency (ZG-25KG Shanghai Huayan, Shanghai, China) induction furnace. The raw materials consisted of Cu, Ni, Si, and Cr (purity 99.5~99.9 at%, TrillionMetals, Beijing, China). The compositions of two alloys were measured using inductively coupled plasma (ICP, ICAP PRO X ICP, Thermo Scientific, Bremen, Germany) analysis, and the results are shown in Table 1. For convenience, the Cu-Ni-Si alloy was designated as 1#, and the Cu-Ni-Si-Cr alloy as 3#.

**Figure 1:**
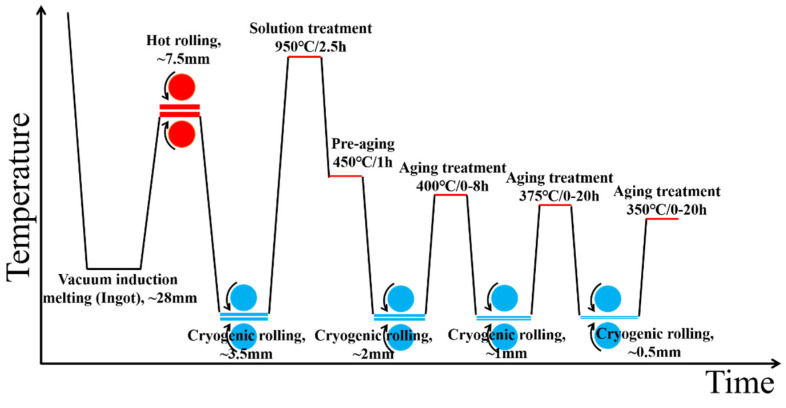
Process flow diagram for two alloy preparation methods.

The ingot was held at 800 °C for 10 min and then hot-rolled, with a deformation of 73.2% achieved. The hot-rolled ingot was ground and cooled in liquid nitrogen for 10 min, followed by cryogenic rolling with a deformation of 53.3%. Subsequently, it was subjected to solution treatment at 950 °C for 2.5 h followed by water quenching, and then pre-aging at 450 °C for 1 h followed by air cooling. The pre-aged samples were subjected to three passes of cryogenic rolling (50% deformation per pass) and subsequent aging treatment, with the parameters presented in Figure 1. The red circle represents a hot-rolled roll, the blue circle represents a cold-rolled roll, and the arrow indicates the direc-tion of roll rotation. The thick red line represents hot-rolled sheet, the thin red line represents heating time, and the blue line represents cold-rolled sheet.

### 2.2. Experimental Methods

The EAM–3A–500 fully automatic micro-Vickers hardness tester, manufactured by Shanghai Hengyi Precision Instrument Co., Ltd. (Shanghai, China), was used to conduct hardness tests in accordance with the GB/T 4340.1–2024 standard [27]. The test load was 200 gf, and the dwell time was 15 s. Thirty valid measurements were obtained for each sample, and the data were sorted in ascending order to characterize the hardness range of the samples. Considering the specific characteristics of the samples, it was unnecessary to calculate an average value in this test; instead, the actual measured data, sorted in ascending order, were used directly to reflect the hardness range of each sample. The test area was selected at a depth of one-quarter of the rolled sheet’s surface. Within a 1 mm × 1 mm area at the center of the rectangular specimen, a micro-Vickers hardness matrix test was conducted with a step size of 71 μm. The test load was set at 50 gf, yielding a total of 225 data points. By plotting a three-dimensional color-mapped surface image with projection, the distribution characteristics of the microhardness regions were analyzed; the variations in hardness reflect the distribution patterns of soft and hard microstructural zones within the alloy.

Using a DDL-50 electronic universal testing machine manufactured by China Machinery Testing Equipment (Jiangsu) Co., Ltd. (Jiangsu, China), tensile property tests were conducted at room temperature in accordance with GB/T 228–2021 [28]. Three parallel samples of each alloy were tested, and the average value was taken as the final result.

Electrical conductivity testing was performed using a SICMATEST 2.070 eddy current conductivity meter manufactured by Hölzle GmbH (Oberreichenbach, Germany). The testing was conducted in accordance with GB/T 32791–2016 [29]. Thirty valid measurements were taken on the same sample; due to the sample’s characteristics, no average value was calculated. Instead, the results were arranged in ascending order to represent the conductivity range of the sample.

The microstructure of the samples was examined using a scanning electron microscope (SEM, JSM–7800F, JEOL, Akishima City, Tokyo, Japan) and a transmission electron microscope (TEM, JEM–F200, JEOL, Akishima City, Tokyo, Japan), with energy-dispersive X-ray spectroscopy (EDS) analysis conducted for compositional characterization. An Empyrean X-ray diffractometer (XRD, PANalytical B.V., Almelo, Overijssel, Netherlands) was used to analyze the phase compositions of the alloys and determine their dislocation densities. The test was conducted at room temperature using CuKα radiation, with a fixed acceleration voltage of 40 kV and an acceleration current of 40 mA. The scanning angle was set between 30° and 95°, at a speed of 1° per minute.

## 3. Results and Discussion

### 3.1. Mechanical Properties and Electrical Conductivity

**Figure 2:**
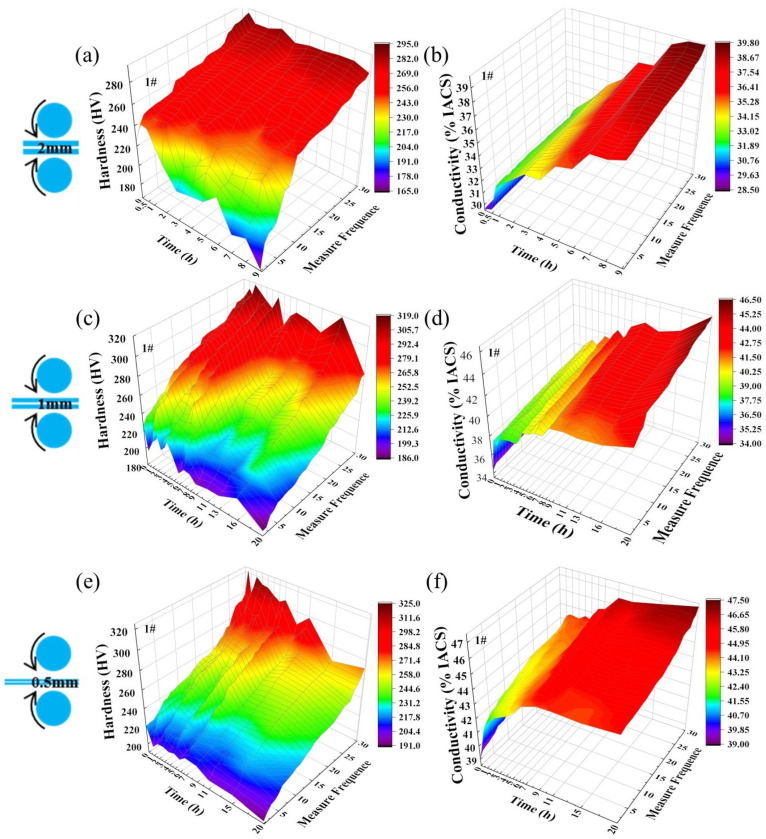
Hardness and electrical conductivity curves of Alloy 1# as a function of cryogenic rolling and aging processes: (**a**,**c**,**e**) hardness; (**b**,**d**,**f**) electrical conductivity.

Figure 2 shows the changes in hardness and electrical conductivity of Sample 1# after three-stage cryogenic rolling and aging treatment. After cryogenic rolling to a thickness of 2 mm, the as-rolled hardness of 1# ranged from 224.2 to 269.6 HV, with electrical conductivity ranging from 29.7 to 30.1%IACS. After aging at 400 °C for 0.5 h, the hardness reached its peak (244.8–291 HV). With increasing aging time, the hardness of the soft microzones decreased continuously, while that of the hard microzones remained largely stable, and the overall electrical conductivity increased. The soft microzones facilitate conductivity due to a sparse precipitation-phase distribution and a relatively pure matrix, while the hard microzones achieve effective precipitation hardening through dense precipitation phases. Balancing strength and conductivity, the aging parameters were determined as 400 °C/8 h. After rolling to 1 mm, the 1# sample exhibited a hardness range of 226.2–292 HV and an electrical conductivity range of 34.4–35.9%IACS. The hard micro-regions’ hardness slightly increased, while the soft micro-regions’ electrical conductivity significantly improved. After aging at 375 °C for 2 h, the hard micro-regions reached their maximum hardness of 301.9 HV. Prolonged aging stabilized the hard micro-region hardness at approximately 290 HV, while the soft micro-region softened noticeably and expanded in proportion. Electrical conductivity continued to increase with extended aging duration. Balancing mechanical and electrical properties, the secondary aging parameters were determined as 375 °C/20 h. After rolling to 0.5 mm, the hardness ranged from 220.5 to 298 HV, with electrical conductivity ranging from 39.1 to 42.1% IACS. A 0.5 h aging at 350 °C reached peak aging (hardness 322.5 HV), with conductivity increasing by only 1%IACS. To balance conductivity improvement and hardness stability, the tertiary aging parameters were selected as 350 °C/7 h. At this point, the alloy hardness ranged from 210.8 to 310 HV, with conductivity approximately 44.1–45.5% IACS.

**Figure 3:**
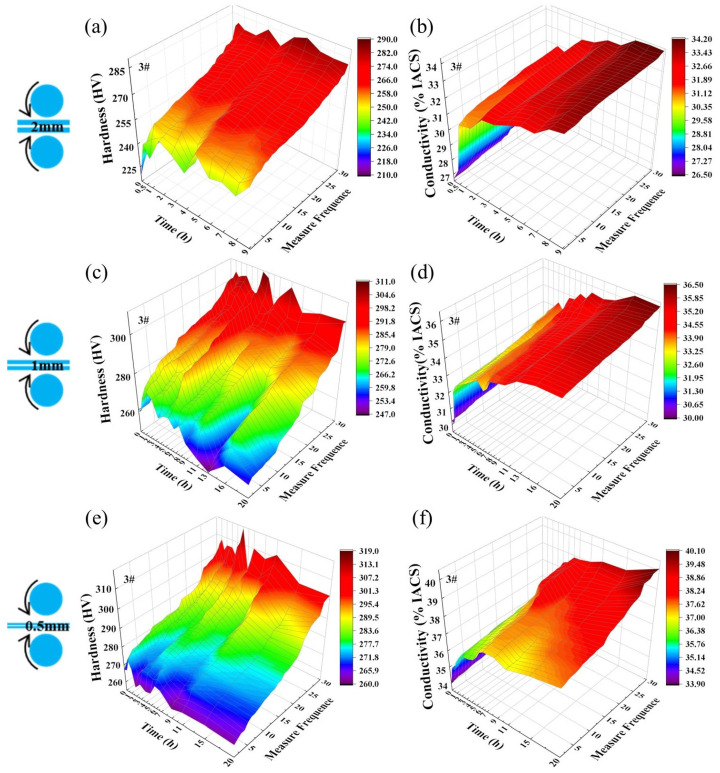
Hardness and electrical conductivity variation curves of Alloy 3# during cryogenic rolling aging processes: (**a**,**c**,**e**) hardness; (**b**,**d**,**f**) electrical conductivity.

Figure 3 shows the changes in hardness and electrical conductivity of the 3# sample after three-stage cryogenic rolling and aging treatments. The as-rolled hardness of 3# ranged from 219.1 to 257.3 HV, slightly lower than that of 1#. After aging at 400 °C for 6 h, the alloy reached peak aging with a hardness of 286.2 HV. Its peak aging time was significantly longer than that of 1#. It indicates that Cr addition promotes Ni_2_Si phase precipitation and delays the aging precipitation process. At peak aging, the 3# sample exhibits a smaller proportion of soft micro-regions, accounting for approximately one-third, demonstrating good compatibility between soft and hard micro-regions. Electrical conductivity initially increases with aging duration before stabilizing. When the aging reaches 8–9 h, both the alloy hardness and electrical conductivity stabilize. Therefore, 400 °C/8 h is selected as the aging parameter. After rolling to 1 mm, the plate hardness increased to 260–279.5 HV. The strength gain in the hard micro-region exceeded that of the 1# sample, indicating rolling more significantly promotes phase precipitation in the 3# sample. During aging at 375 °C, the soft micro-region exhibited noticeable softening, while the hard micro-region stabilized at approximately 300 HV. The conductivity growth trend gradually slowed, reaching about 35.2–36.3% IACS after 20 h of aging. Thus, the secondary aging parameters were determined as 375 °C/20 h. After rolling to 0.5 mm, the hardness ranged from 267.2 to 293.5 HV, showing further improvement over the previous stage, indicating that the 3# sample still possesses precipitation potential. Peak aging was reached at 350 °C after 5 h, with a hardness peak of 318 HV, stabilizing around 310 HV after 9 h of aging. Electrical conductivity reached an increase bottleneck after 9 h. Balancing mechanical and electrical performance requirements, the optimal aging parameters were determined as 350 °C/9 h. At this condition, the alloy hardness ranged from 260 to 312 HV, with electrical conductivity ranging from 37.2 to 39.1% IACS.

Compared with the 1# sample, the 3# sample showed no significant increase in the hardness of hard micro-regions, but the hardness of its soft micro-regions was significantly enhanced, with a more uniform hardness distribution. This is because Cr addition promotes finer precipitation phase formation, while cryogenic rolling induces dislocations that inhibit their movement, thereby boosting alloy strength and hardness. Additionally, the soft micro-regions accounted for less than one-third of the three-stage rolled state in the 3# sample, and their diffusion extent remains lower than that of the 1# sample even with extended aging. The smaller proportion of soft micro-regions led to a slight decrease in electrical conductivity.

Tensile tests were conducted on samples of sample 1# and 3# rolled to 1 mm and 0.5 mm under optimal aging conditions, and the results are shown in Table 2. Under the identical deformation heat treatment control processes (when rolled to 1 mm), the addition of Cr significantly enhanced the tensile strength and yield strength of the Cu-Ni-Si alloy, exhibiting higher tensile strength (816 MPa) and yield strength (775 MPa). When rolled to 0.5 mm, both alloys exhibited strength improvements. The 3# sample achieved tensile strength and yield strength of 862 MPa and 818 MPa, respectively, representing 13% and 23% increases over the 1# sample. The addition of Cr promotes precipitation phase formation and pinning of dislocations, leading to significant strength improvement. However, due to cumulative deformation exceeding 85% from multiple cryogenic rolling passes, both alloys had a relatively high dislocation density, resulting in low plasticity. These results clearly demonstrate that adding trace amounts of Cr effectively modulates the comprehensive properties of Cu-Ni-Si alloys, particularly highlighting the strengthening effect of Cr.

### 3.2. Effects of Cr on the Microstructure of Cu-Ni-Si Alloy

**Figure 4:**
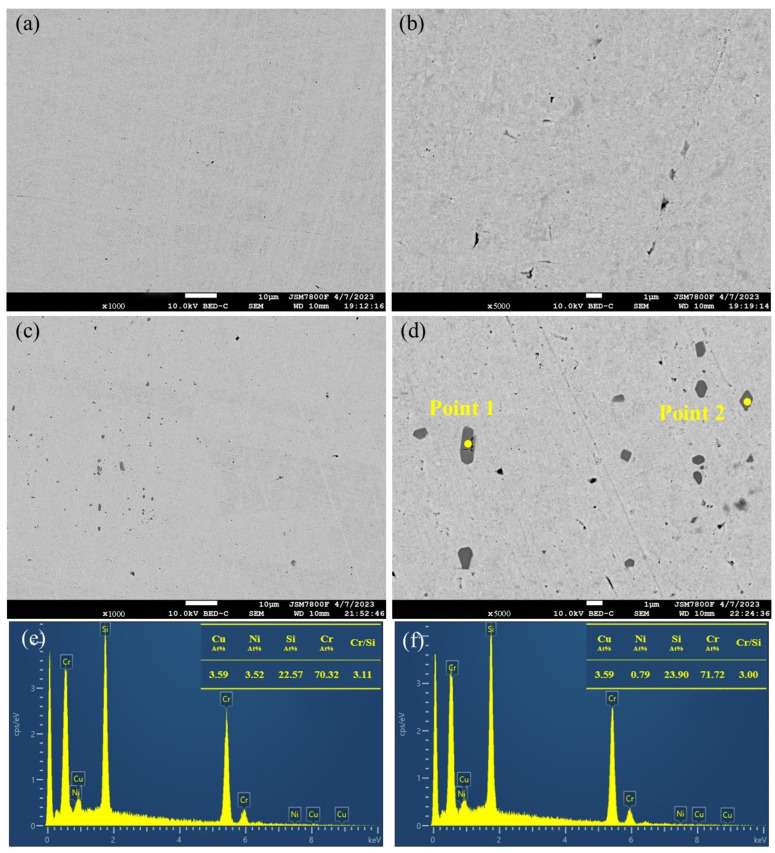
SEM results after solution treatment at 950 °C/2.5 h for two alloys (**a**,**b**) 1#; (**c**,**d**) 3#; (**e**) Scan results for Point 1; (**f**) Scan results for Point 2.

Figure 4a,b shows the SEM morphology of sample 1# after solution treatment. Ni and Si were completely dissolved back into the grains and grain boundaries, with no obvious element segregation or residual phases. In the sample with added Cr (Figure 4c–f), Ni was almost entirely dissolved back into the matrix, but a few residual phases remained with non-uniform sizes. Point scan results confirmed these as Cr_3_Si phases, which formed during melting and casting and persisted after solution treatment.

**Figure 5:**
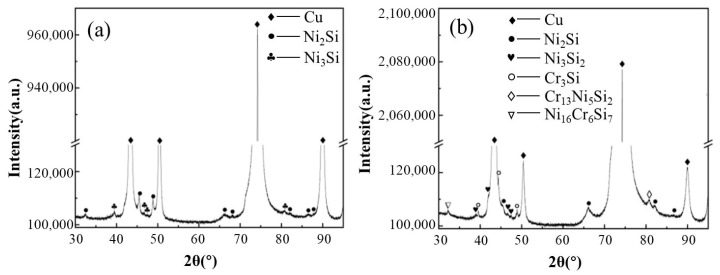
XRD patterns of two alloys under optimal conditions of three-stage deformation heat treatment (**a**) 1#; (**b**) 3#.

Figure 5 shows the XRD patterns of the two alloys in their optimal states after three-stage thermomechanical processing. In addition to the Cu matrix, the 1# sample showed distinct peaks for Ni_2_Si and Ni_3_Si phases. Compared with the 1# sample, the addition of Cr in 3# induced a competitive precipitation effect between Cr and Ni for Si. Benefiting from the stronger thermodynamic affinity between Cr and Si, the Cr_3_Si phase formed in the alloy. In the calibration analysis of the precipitation phases, the Ni/Si atomic ratio showed a decreasing trend, while multiple Cr-containing Ni-Si-Cr ternary precipitation phases also formed in the alloy.

**Figure 6:**
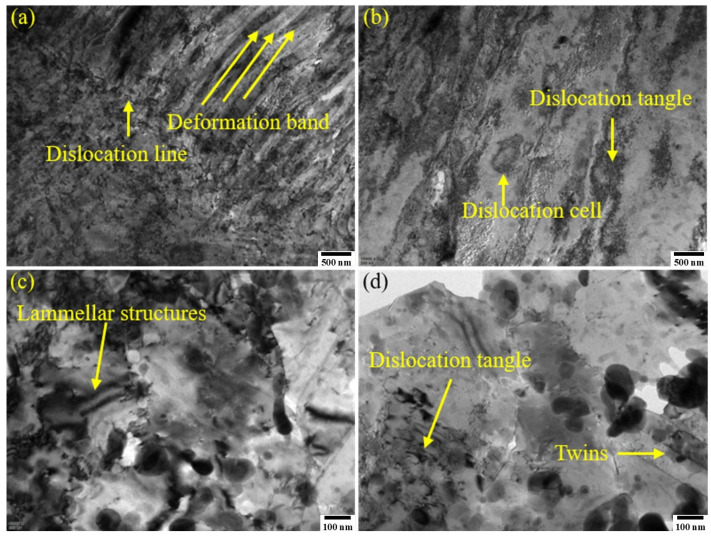
TEM images of the optimal state of the three-stage deformation heat treatment for sample 1#: (**a**,**b**) deformation zone microstructure and dislocation morphology; (**c**) layered structure morphology; and (**d**) twin morphology.

**Figure 7:**
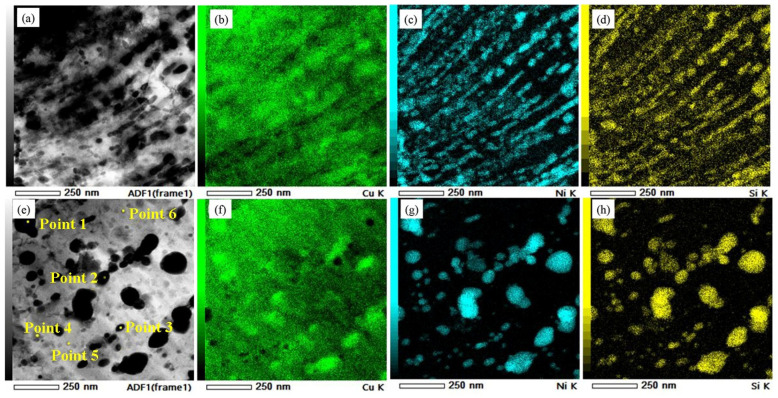
Phase analysis of optimal precipitation phases in the three-stage deformation heat treatment of sample 1#: (**a**) precipitation phase morphology distributed along deformation bands; (**b**–**d**) face scan results corresponding to (**a**); (**e**) multiscale phase morphology; and (**f**–**h**) face scan results corresponding to (**e**).

TEM and EDS characterizations were performed on both alloys in their optimal states. Figure 6 shows the TEM morphologies of the 1# sample. At low magnification, the alloy grains appeared distinctly elongated, with numerous deformation bands, dislocation lines, dislocation cells, and dislocation tangles present internally. A large number of precipitates pinned the dislocation-dense regions. At high magnification, distinct twins and layered structures were observed. The matrix contained numerous disk-shaped precipitates of varying sizes. Precipitates around 50 nm in size were concentrated at grain boundaries, with fine precipitates effectively pinning dislocations. EDS analyses of the deformation bands and intragranular precipitates are shown in Figure 7. Approximately 50 nm precipitates on the deformation bands were interconnected, forming 250 × 50 nm stripes enriched in Ni and Si. Dislocations, twins, and deformation bands introduced by cryogenic rolling provided nucleation sites for precipitates, and aging provided the precipitation driving force, promoting Ni and Si element precipitation. Increased rolling deformation refined the deformation bands, effectively suppressing Ni_2_Si phase coarsening. At the same magnification, intragranular precipitates exhibited diverse sizes (Figure 7e). EDS point scans were performed on the precipitates classified by size, with elemental atomic ratios shown in Table 3. The approximately 200 nm phases were Ni_3_Si; rolling promoted the precipitation of Ni and altered the Ni/Si atomic ratio. All phases in the 20 nm–100 nm size range were Ni_2_Si. The aging time for 1# was not set at the peak aging point. To ensure electrical conductivity after reaching the peak aging state, the holding time was extended. This caused the existing Ni_2_Si phases in the matrix to coarsen and grow. However, small-sized Ni_2_Si phases still precipitated. Consequently, the strength properties of the 1# sample did not decrease significantly immediately after entering the over-aged state.

**Figure 8:**
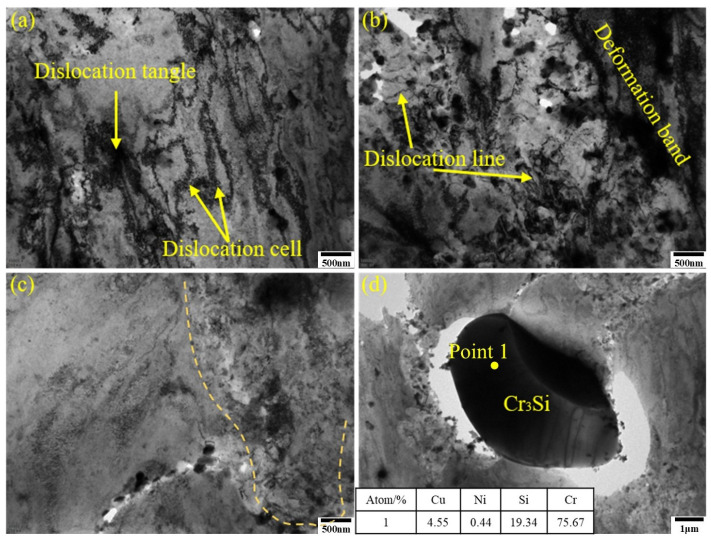
TEM images of the optimal state of the three-stage deformation heat treatment for sample 3#: (**a**) dislocation patterns; (**b**) deformation zone microstructure; (**c**) contrasting morphology of dense and sparse dislocation tangles; and (**d**) coarse Cr_3_Si morphology.

**Figure 9:**
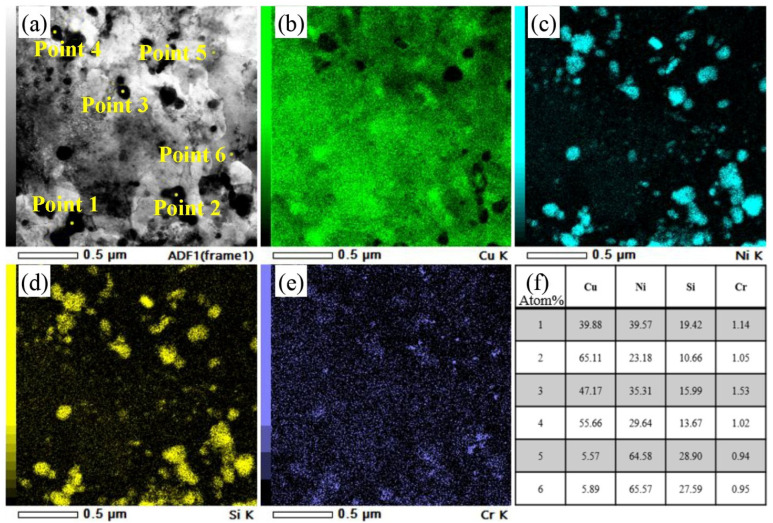
Multiscale phase energy spectrum analysis of the optimal condition for the three-stage deformation heat treatment of 3#: (**a**) multiscale precipitation phase morphology map; (**b**–**e**) surface scanning results corresponding to (**a**); and (**f**) d ratios of selected elements at specific points.

Figure 8 shows the TEM microstructure of the 3# sample after aging. At low magnification, dislocation cells, dislocation lines, and dislocation tangles were observed. Multiple rolling passes fragmented and refined the dislocation lines, which radiated around phase boundaries. Deformation bands were also present in the alloy. Compared with the 1# sample, 3# exhibited distinct phase-dislocation dense zones and dislocation-free sparse zones (as shown on either side of the dashed line in Figure 8c). The dense zones were primarily distributed at grain boundaries and subgrain boundaries. The matrix contained micron-sized Cr-rich phases measuring 7 × 4 μm. EDS analysis of Point 1 in Figure 8d confirmed these as Cr_3_Si phases, which were residual phases remaining after solution treatment at 950 °C/2.5 h. These phases formed through fragmentation during multiple rolling passes and subsequent aging coarsening. Due to their large size, they tended to detach during transmission double-jet polishing. Nanoscale precipitation clusters were visible around their periphery. In the 3# sample, Cr_3_Si phase sizes showed polarized distribution: one type consisted of micron-sized Cr_3_Si prone to grain boundary segregation after 950 °C solution treatment, which refined into elongated structures along the rolling direction after multi-pass rolling fragmentation. The other type comprised fine Cr_3_Si phases (approximately 10 nm) precipitated during aging, enhancing matrix purity and improving electrical conductivity. EDS analysis of the phase-dense region (Figure 9) revealed multi-scale precipitates ranging from 10 to 200 nm. Size-statistical elemental atom ratios indicated Ni/Si ≈ 2, confirming the presence of Ni_2_Si phase. The fine Ni_2_Si phases primarily contributed to strengthening effects, while the coarse Ni_2_Si phase formed through growth of earlier precipitation phases during rolling and aging. The EDS mapping results also revealed localized Cr segregation in the edges of Ni_2_Si phases. No distinct Ni_3_Si phase was observed in the alloy, attributed to trace Cr promoting Ni_2_Si formation while competing with Ni for Si, thereby reducing the Ni/Si ratio. The formation of multi-scale precipitates originated from the growth of early precipitates during rolling and aging. After exceeding the peak aging point, the alloy’s strength exhibited only minor fluctuations rather than a significant decline. This is because new, fine Ni_2_Si strengthening phases continued to precipitate at dislocations, subgrain boundaries, and other defects under the aging precipitation driving force, counteracting the performance degradation caused by the coarsening of earlier precipitates.

### 3.3. Effects of Cr Elements on the Strengthening Mechanism

The strengthening mechanisms of Cu-Ni-Si alloys primarily rely on age hardening, supplemented by solution strengthening, grain refinement strengthening, and work hardening. In this study, the combination of multi-pass rolling and aging processes mainly depends on age hardening and work hardening, with the degree of work hardening determined by the dislocation density in different alloy conditions. Therefore, the yield strength formation mechanism of the experimental alloy under three-stage cryogenic rolling and aging treatments primarily involves precipitation strengthening and dislocation strengthening.

#### 3.3.1. Dislocation Strengthening

Macroscopic quantitative determination of dislocation density requires XRD testing using an X-ray diffractometer, with the alloy’s dislocation density calculated according to Equation (1) [30]:

(1) $$∆K=\left(\frac{0.9}{D}\right)+{\left(\frac{\pi Tb^{2}}{2}\right)}^{0.5}\rho^{0.5}K^{2}{\bar{C}}_{hkl}^{0.5}$$

where Δ*K* denotes the strain-related component of the maximum full width at half maximum (FWHM) in reciprocal space, expressed as Δ*K* = (2* cosθ∙*Δ*θ)*/*λ*; *K* = 2 *sinθ*/*λ*; *θ* represents the diffraction angle; *λ* is the X-ray wavelength, typically taken as 1.5405 Å; *D* corresponds to the crystallite size; *T* is the Wilkens rearrangement parameter, dependent on the effective radius of the cut-through dislocations; *b* denotes the Burgers vector with a magnitude of 0.2556 nm; ρ indicates the dislocation density; and ${\bar{C}}_{hkl}$ represents the average contrast factor for each specific crystallographic plane (hkl).

The ${\bar{C}}_{hkl}$ values for screw dislocations and edge dislocations differ significantly. When multiple dislocation types coexist in an alloy, an average value must be taken for approximate calculations. According to Equation (1), the dislocation density is proportional to the slope of the ∆*K-K* curve.

**Figure 10:**
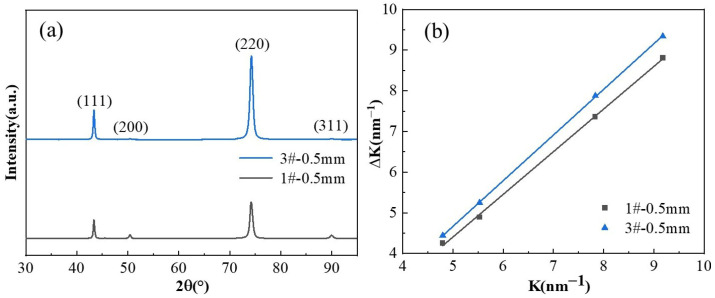
XRD spectra and dislocation densities of two alloys aged at 350 °C peak: (**a**) XRD spectra of the two alloys; (**b**) dislocation densities of two alloys.

Figure 10 shows the XRD patterns and ∆*K-K* curves for two alloy plates at 350 °C after different aging times at a thickness of 0.5 mm. The figures reveal that the dislocation density of 3# is higher than that of 1#. Rolling deformation increases the dislocation density in alloys. For age-hardening alloys, the introduction of dislocations affects the precipitation driving force and nucleation sites, macroscopically manifesting as an increase in yield strength with increasing deformation. Under identical deformation heat treatment conditions, the dislocation density introduced by cryogenic rolling should theoretically be comparable. However, calculations indicate a higher dislocation density in the 3# sample. This suggests that the addition of chromium not only promotes the formation of the Cr_3_Si phase but also enhances Ni_2_Si precipitation while suppressing Ni_3_Si formation. This process intensifies dislocation pinning and stacking effects, increasing dislocation density and consequently boosting alloy strength.

#### 3.3.2. Precipitation Strengthening

**Figure 11:**
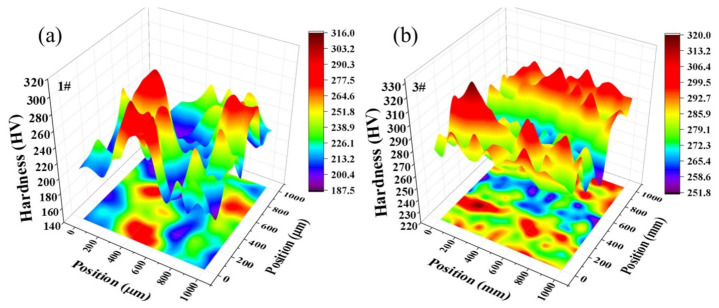
Hardness matrix of two alloys at different aging times at 350 °C: (**a**) 1#; (**b**) 3#.

The hardness matrix distributions for both alloys are shown in Figure 11. The 1# sample exhibited an excessively low proportion of hard micro-regions, which tended to be enveloped by soft micro-regions. In this case, electrical conductivity was facilitated through the relatively pure matrix of the soft micro-regions, resulting in good electrical conductivity. The 3# sample demonstrated a well-coordinated distribution of soft and hard micro-regions, exhibiting an elongated trend along the rolling direction of the sheet and achieving the highest strength.

Since the manufacturing processes for both alloys are identical, the variation in micro-hardness distribution stems from differences in the original microstructure after 2.5 h of solution treatment at 950 °C, caused by the addition of chromium. For the 1# sample, all elements were fully dissolved back into the matrix after 2.5 h solution treatment. The 3# sample contained a small amount of Cr_3_Si phases, which enhanced electrical conductivity and further refined the microstructure during subsequent rolling. Transmission electron microscopy reveals distinct dislocation bypassing around the precipitated phases, indicating that the Orowan bypass mechanism dominates the strengthening effect in this alloy.

The aging process causes solute atoms in the supersaturated solid solution to diffuse within the matrix, nucleating and growing at defects such as dislocations, subgrain boundaries, and grain boundaries to form strengthening phases. TEM morphological analysis revealed that most dislocation lines pinned the precipitation phases and exhibited a radial state, making it easier for dislocations to bypass the precipitation phases. Therefore, the primary strengthening mechanism during aging is the Orowan bypass mechanism. The various influencing factors of age hardening are summarized in Equation (2) [31,32].

(2) $$∆\sigma_{or}=0.81\frac{MGb}{{2\pi \left(1-\upsilon \right)}^{1/2}}\frac{\ln\left(d_{p}/b\right)}{\lambda -d_{p}}$$

In the equation, *M* represents the Taylor factor, taken as 3.06; *G* denotes the shear modulus of the matrix, with 44 GPa for the Cu matrix; $d_{p}$ is the average diameter of the second-phase particles; *ν* is Poisson’s ratio, taken as 0.3; *b* is the dislocation brachistochrone vector; and *λ* is the distance between particles, which can be calculated using Equation (3) [33,34].

(3) $$\lambda =d_{p}\sqrt{\frac{3\pi }{8f_{v}}}$$

In the equation, $f_{v}$ represents the volume fraction of the precipitated phase. According to Equation (2), the increase in strength is inversely proportional to the distance λ between the precipitated phases. Observing particle calculation Formula (3), it is found that *λ* is directly proportional to $d_{p}$ and inversely proportional to ${f_{v}}^{1/2}$. Therefore, the smaller the size of the precipitated phase and the denser its distribution, the more pronounced the effect of the dislocation bypass strengthening mechanism [35].

The volumetric fraction calculation expression for the precipitated phase is shown in Equation (4).

(4) $$f=\frac{V_{p}}{V_{p}+V_{Cu}}$$

The ratio of the actual volume precipitated under the selected aging parameters for the δ-Ni_2_Si phase to the equilibrium volume achieved at a fixed aging temperature is defined as the precipitation ratio *X* at the selected state:

(5) $$X=\frac{V_{p}}{V_{p-MAX}}$$

In the equation, $V_{p-MAX}$ denotes the thermodynamic equilibrium volume, i.e., the maximum volume of precipitated phase within a given volume at a fixed aging temperature; $V_{p}$ represents the volume of precipitated phase per unit volume during a selected aging time.

According to Matthiessen’s law, electrical conductivity exhibits a linear relationship with the volume fraction of the second phase precipitated during aging. Therefore, the conductivity formula for a given aging time is expressed as Equation (6).

(6) $$C=C_{0}+\left(C_{MAX}-C_{0}\right)X$$

In the equation, *C*_0_ represents the initial electrical conductivity at the current aging stage, while $C_{MAX}$ denotes the maximum achievable conductivity. Therefore, the precipitation ratio can be theoretically calculated based on the final-state conductivity. However, these calculations are entirely based on the assumption of uniform precipitation. For this series of alloys, they can serve as theoretical estimates, as the alloy microstructure itself is inherently non-uniform, featuring varying degrees of soft and hard micro-regions coexisting.

For the 1# sample, the precipitation phases consist of Ni_2_Si and Ni_3_Si phases. TEM morphology characterization indicates that the precipitation phase size in the 1# sample is around 50 nm, but the number per unit area is significantly lower than that in the 3# sample. Consequently, the precipitation strengthening effect in Alloy 1 is relatively low. For the 3# sample, the precipitation phase size exhibits a wider range, including phases around 50 nm and phases below 10 nm. The volume fraction of multiple phases is higher than that of the 1# sample. The combination of multi-scale phases leads to a more pronounced increase in precipitation strengthening, resulting in higher strength.

## 4. Conclusions

This paper systematically reveals the patterns and mechanisms by which Cr addition affects the microstructure and properties of Cu-Ni-Si alloys, providing more universally applicable design strategies for developing Cu-Ni-Si alloys that combine high strength, high electrical conductivity, and good uniformity. The main conclusions are as follows:

1. After triple-stage cryogenic rolling and aging treatment, the Cu-Ni-Si-Cr (3#) alloy achieves a tensile strength of 862 MPa, a yield strength of 818 MPa, and electrical conductivity reaching 37.2–39.1% IACS. Compared to the Cu-Ni-Si (1#) alloy, the 3# alloy exhibits a 13% increase in tensile strength and a 23% increase in yield strength, with a slight decrease in electrical conductivity.
2. The precipitation behavior differs between the 1#and 3# samples. Both alloys form multi-scale nanoscale δ-Ni_2_Si phases, but the 1# sample additionally produces nanoscale Ni_3_Si phases, and the 3# sample produces micrometer/nanoscale Cr_3_Si phases. Cr addition induces competitive precipitation between Cr and Ni for Si, suppressing Ni_3_Si formation while refining grains and inhibiting δ-Ni_2_Si growth, thereby enhancing mechanical properties.
3. Cr addition promotes precipitation of the nano-scale strengthening phase Ni_2_Si and formation of the Cr_3_Si phase, yielding the most pronounced precipitation strengthening effect. Cr addition accelerates dislocation proliferation, increasing nucleation rate while reducing the size of Ni_2_Si precipitates, thereby enhancing alloy mechanical properties. The 3# sample exhibits alternating soft/hard microzones with staggered distribution, resulting in more uniform overall performance. However, soft microzones remain scarce, leading to a slight decrease in alloy electrical conductivity.

## Figures and Tables

**Table 1:** Chemical composition of Cu-Ni-Si and Cu-Ni-Si-Cr alloys.

Alloy	Chemical Constituents (at%)			
	Ni	Si	Cr	Cu
Cu-Ni-Si	3.45	1.68	/	Balanced
Cu-Ni-Si-Cr	3.48	1.61	0.33	Balanced

**Table 2:** Optimal aging conditions for the tensile properties of the 1# and 3# alloy plates at different thicknesses.

Alloy	Tensile Strength (MPa)	Yield Strength (MPa)	Elongation (%)
1#-1 mm	705 ± 2.8	617 ± 3.2	3.9 ± 0.29
3#-1 mm	816 ± 2.9	775 ± 3.8	1.9 ± 0.32
1#-0.5 mm	763 ± 3.6	667 ± 3.5	3.2 ± 0.23
3#-0.5 mm	862 ± 2.8	818 ± 2.5	2.3 ± 0.25

**Table 3:** Elemental atomic ratios in point-by-point spectral analysis of sample 1#.

Atom%	Cu	Ni	Si	Ni/Si
1	46.20	41.52	12.28	3
2	29.88	48.15	21.97	2
3	57.95	29.64	12.41	2
4	98.97	0.74	0.30	2
5	32.60	46.26	21.14	2
6	99.17	0.54	0.29	2

## Data Availability

The original contributions presented in this study are included in the article. Further inquiries can be directed to the corresponding author.
